# Cluster of differentiation antigens: essential roles in the identification of teleost fish T lymphocytes

**DOI:** 10.1007/s42995-022-00136-z

**Published:** 2022-08-19

**Authors:** Hong-fei Tian, Jing Xing, Xiao-qian Tang, Heng Chi, Xiu-zhen Sheng, Wen-bin Zhan

**Affiliations:** 1 Laboratory of Pathology and Immunology of Aquatic Animals, KLMME, Fisheries College, Ocean University of China, Qingdao, 266003 China; 2 Laboratory for Marine Fisheries Science and Food Production Processes, Pilot National Laboratory for Marine Science and Technology (Qingdao), Qingdao, 266237 China

**Keywords:** T lymphocytes, Surface marker, Classification, Monoclonal antibody, Fish

## Abstract

Cluster of differentiation (CD) antigens are cell surface molecules expressed on leukocytes and other cells associated with the immune system. Antibodies that react with CD antigens are known to be one of the most essential tools for identifying leukocyte subpopulations. T lymphocytes, as an important population of leukocytes, play essential roles in the adaptive immune system. Many of the CD antigens expressed on T lymphocytes are used as surface markers for T lymphocyte classification, including CD3, CD4 and CD8 molecules. In this review, we summarize the recent advances in the identification of CD molecules on T lymphocytes in teleosts, with emphasis on the functions of CD markers in the classification of T lymphocyte subsets. We notice that genes encoding CD3, co-receptors CD4 and CD8 have been cloned in several fish species and antibodies have been developed to study protein expression in morphological and functional contexts. T lymphocytes can be divided into CD4^+^ and CD8^+^ cells discriminated by the expression of CD4 and CD8 molecules in teleost, which are functionally similar to mammalian helper T cells (Th) and cytotoxic T cells (Tc), respectively. Further studies are still needed on the particular characteristics of teleost T cell repertoires and adaptive responses, and results will facilitate the health management and development of vaccines for fish.

## Introduction

Leukocytes express distinct assortments of molecules on their cell surfaces. These surface molecules play critical roles in signaling and many reflect different stages of leukocytes lineage-specific differentiation (Cruse et al. 2004). Antibodies that react with cell surface antigens are known to be one of the most essential tools for identifying leukocyte subpopulations. Therefore, in the early years, large numbers of monoclonal antibodies (mAbs) that react to these cell surface molecules were developed by immunologists, each with different associated nomenclatures (Zola et al. 2005). In the absence of a unified nomenclatural system for mAbs, it was very difficult to tell if more than one antibody was specific for the same molecule (Zola et al. 2003). In the 1980s, the establishment of the human leukocyte differentiation antigens (HLDA) workshop brought order to the chaos (Bernard and Boumsell 1984; Boumsell 1996). A standard nomenclature for several mAbs that react with a specific antigen has been implemented, which provides consistency and uniformity when referring to the same molecules (Engel et al. 2015). Clusters of antigens on the surface of leukocytes can be clearly designated by their reactions with mAbs. This designation of antigens is called clusters of differentiation (CDs), and this common nomenclature has been applied not only to human, but also to other vertebrates, including teleost fish. To date, more than 400 human proteins have been designated as CD markers, and the HLDA workshops continue to be held on a 4-year cycle with the main purpose of updating newly characterized molecules (Zola et al. 2005).

T lymphocytes, as an important population of leukocytes, play essential roles in the adaptive immune system. Many of the CD antigens expressed on T lymphocytes are involved in signal transduction and activation, and several CD antigens can be used as cell surface markers of T lymphocyte classification, including CD3, CD4, CD8, CD28 and CTLA4 (CD152) (Nakanishi et al. 2015). For example, CD3 and TCR molecules coexist on the surface of T cells, forming the basic structure of T cell antigen-specific recognition and cell activation signal transmission (Ashwell and Klausner 1990; Klausner et al. 1990). T lymphocytes can be divided into T helper (Th) and T cytotoxic (Tc) cells, which are distinguished by the expression of CD4 and CD8 glycoproteins, respectively (Kato et al. 2013). The counter-receptors of CD28 and CTLA4 are important T cell costimulatory receptors and involved in the activation or inactivation of T cells (Chen and Flies 2013). Teleost fish, the oldest vertebrate group, exhibit all the major features of the mammalian immune system and have both innate and adaptive immunity. T lymphocytes and their subsets have been found and identified in fish. According to the definition and nomenclature of CD antigens in mammals, many of CD molecules expressed on T lymphocytes also have been cloned and identified in multiple fish species (Castro et al. 2011). Together with the development of mAbs that recognize surface molecules on T lymphocytes, the phenotypes and properties of T cells have become important issues for fish immunologists. Two subpopulations, CD4^+^ and CD8^+^ T lymphocytes (functionally identified as Th and Tc cells in mammals) have also been found and characterized in fish (Nakanishi et al. 2015). Furthermore, CD2, CD28, CTLA4 and other cluster differentiation antigens have also been characterized in teleost fish (Bernard et al. 2007; Cho et al. 2017; Hu et al. 2012; Jeswin et al. 2017; Shao et al. 2018). However, the precise roles of fish CD antigens in the classification, signal transduction and activation of T cells are still unclear.

In this review, we summarize recent progress in the identification of CD molecules on T cells in teleost fish, with emphasis on the functions of CD markers in the classification of fish T lymphocyte subsets. The main aims are to deepen our understanding of the precise role of fish T lymphocyte subpopulations in adaptive immunity, and to facilitate the health management and development of vaccines for fish.

## CD3 subunits as specific markers for T lymphocytes in fish

### Molecular characterization of the CD3 complex

**Fig. 1:**
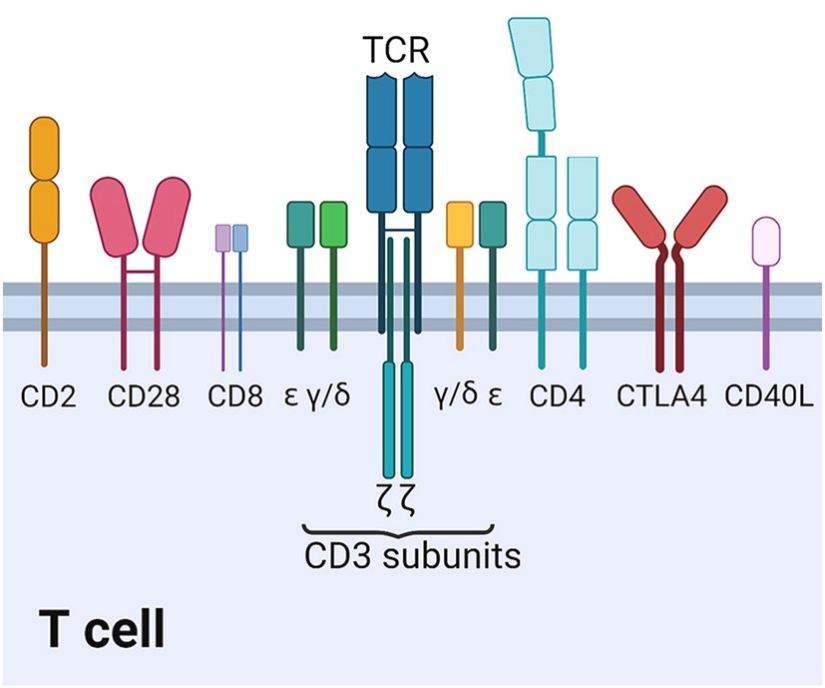
Cluster of differentiation antigens expressed on T lymphocytes in teleost fish. According to the definition and nomenclature of CD antigens in mammals, many of CD molecules expressed on T lymphocytes have also been cloned and identified in multiple fish species. For example, CD3 and TCR molecules coexist on T cells, forming the basic structure of T cell antigen-specific recognition and cell activation signal transmission. T lymphocytes can be divided into T helper (Th) and T cytotoxic (Tc) cells, distinguished by the expression of CD4 and CD8 glycoprotein, respectively. The counter-receptors of CD28 and CTLA4, as important T cell costimulatory receptors, are involved in activation or inactivation of T cells. CD2, CD40L and other cluster differentiation antigens are also characterized in teleost fish

CD3 molecules coexist in the form of TCR-CD3 complex on T lymphocytes, and this complex is made up of an αβ or γδ heterodimer of TCR and the subunits (γ, δ, ɛ and ζ chains) of CD3 (Kim and Park 2005; Park et al. 2001). The TCR-CD3 complex plays essential roles in specific antigen recognition, cell activation and signal transmission in T cells (Jung et al. 2017; Kuhns et al. 2006). Structurally, the γ, δ and ɛ chains of CD3 are members of the immunoglobulin (Ig) superfamily and consist of an extracellular Ig-like domain, a transmembrane helix and a cytoplasmic tail, whereas the CD3ζ chain has a short extracellular peptide, a transmembrane part and a long cytoplasmic tail (Liu et al. 2008). All the CD3 subunits contain immunoreceptor tyrosine-based activation motifs (ITAMs) in the intracellular domains that connect with tyrosine kinases during the signal transduction (Kuhns et al. 2006; Randelli et al. 2011). Similar to mammals, four T cell receptor genes, which encode the TCR α-, β-, γ- and δ-chains, and three CD3 chains (CD3γ/δ, -ɛ and -ζ), have been cloned in teleost fish (Fig. 1), thereby displaying the conservation of functional characteristics (Langenau and Zon 2005; Maisey et al. 2011). The CD3γ/δ gene that corresponds to the forerunner of mammalian CD3γ and CD3δ genes has been reported in multiple fish species, as well as from birds and amphibians, suggesting a common ancestor of mammalian CD3γ and CD3δ (Araki et al. 2005; Kim and Park 2005; Liu et al. 2008; Park et al. 2001; Randelli et al. 2011; Shang et al. 2008).

## Antibodies used to identify CD3^+^ T lymphocytes

**Fig. 2:**
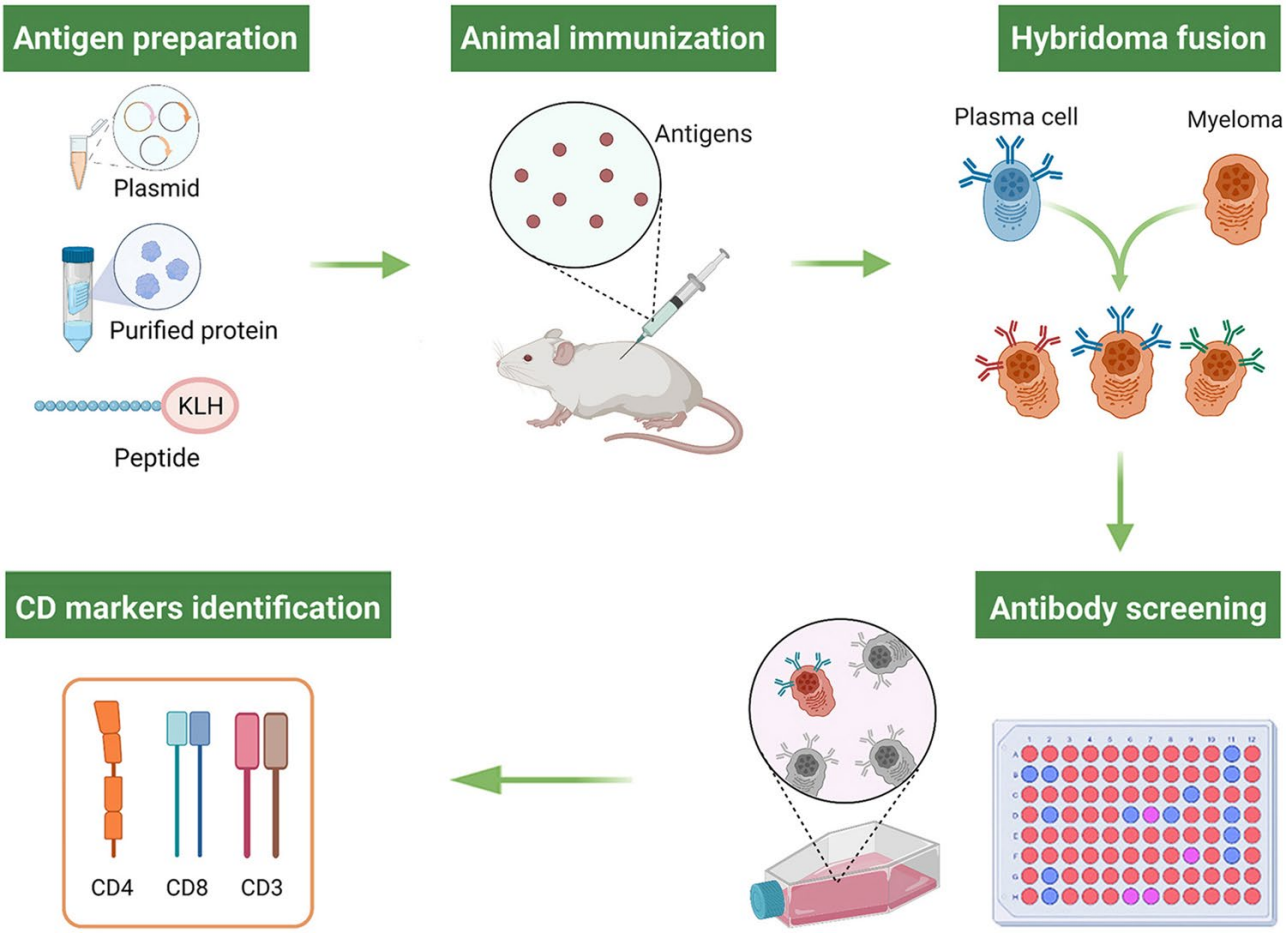
Development of monoclonal antibodies against CD marker molecules in fish. For the selection of immunogens, the eukaryotic plasmids, purified recombinant proteins or peptide-KLH complexes are used as antigens. After immunization of mouse, splenocytes and myeloma cells are fused and then the positive hybridoma cells are immunologically screened. The positive hybridoma cells were expanded and cultured, and the cell supernatant was collected as monoclonal antibody. The monoclonal antibodies against CD molecules are important tools for the identification of T cell subsets in fish

In mammals, the TCR-CD3 complex has been the focus of intense research covering aspect of biosynthesis, assembly, structure and signaling, which has given insights into the roles of T cells in immunity (Göbel and Dangy 2000). Although the CD3 subunits have been reported in multiple fish species, the research was focused on gene cloning rather than structure or signaling (Kim and Park 2005). However, CD3 molecules are essential cell surface markers of T lymphocytes, and the antibodies against these molecules are powerful tools to identify T lymphocytes and study their immune properties in fish. In earlier studies, anti-human CD3 antibodies have been used to react with CD3 molecules in different mammalian, avian and even fish species, when the sequence information of non-mammalian CD3 proteins were not available (Bertram et al. 1996; Cook et al. 2001; Keresztes et al. 1996; Wilkinson et al. 1995). ZAP70, a tyrosine kinase protein (70 kDa), expressed in T cells and crucial for their selective activation, has also been used to label fixed T cells in fish by using anti-human ZAP70 mAbs (Yoon et al. 2015). With the need of research and development of sequencing, mAbs that specifically recognized fish T cells have been developed (Fig. 2). The mAbs of DLT15 were developed that recognize thymocytes and T lymphocytes in peripheral tissues of sea bass (*Dicentrarchus labrax*), and the mAbs of WCL38 were produced to recognize intestinal T cells in the common carp (*Cyprinus carpio*) (Rombout et al. 1998; Scapigliati et al. 2000). The antibodies or RNA probes against TCR molecules were also used to label T lymphocytes in fish (Picchietti et al. 2008; Romano et al. 2011; Timmusk et al. 2003). In addition, the CD3 subunit complex is specifically expressed on the surface of T cells, and the generation of antisera or mAbs against fish CD3 chains are powerful tools for identifying T cells (Table 1). In salmon, plenty of CD3ɛ^+^ T cells in the thymus, intestine and gill were identified using the antisera against a synthetic peptide of the CD3ɛ chain (Koppang et al. 2010). Subsequent morphological analysis revealed that T cells aggregated in the thymus, spleen, and even in the interbranchial lymphoid tissue of salmon. The results suggest that the interbranchial lymphoid tissue in fish is an important location of T cell aggregation and for facilitating the encounter of antigens (Koppang et al. 2010).

**Table 1:** The antibodies against to teleost fish CD3, CD4 and CD8 molecules, respectively

Marker molecules	Fish species	Type of antibody	Application	References
CD3ɛ	Salmon	pAb	WB, FCM, IHC, IFA	Koppang et al. (2010)
	Trout	mAb	Co-IP, FCM, IFA	Boardman et al. (2012)
	Salmon	pAb	WB, FCM, IFA	Maisey et al. (2016)
	Flounder	pAb	Co-IP, FCM, IFA, IHC	Tang et al. (2017)
	Flounder	mAb	WB, FCM, IFA, FACS	Jung et al. (2017)
CD4-1	Spotted green pufferfish	pAb	WB	Wen et al. (2011)
	Ginbuna crucian carp	mAb	WB, FCM, IFA, FACS	Toda et al. (2011)
	Japanese pufferfish	pAb	WB, FCM, IFA, FACS	Kono and Korenaga (2013)
	Zebrafish	pAb	WB, IFA, IHC, FCM, FACS	Yoon et al. (2015)
	Trout	mAb	WB, FCM, IFA, FACS	Takizawa et al. (2016)
	Trout	pAb	WB, FCM, IFA, FACS	Maisey et al. (2016)
	Flounder	pAb mAb	WB, FCM, IFA, IHC, FACS WB, FCM, IFA, FACS	Xing et al. (2017a, b) Xing et al. (2020)
	Ginbuna crucian carp	pAb	IFA	Kato et al. (2019)
	Flounder	mAb	WB, FCM, IFA, FACS	Jung et al. (2020a)
CD4-2	Spotted green pufferfish	pAb	WB, FCM, IHC, FACS	Wen et al. (2011)
	Trout	mAb	WB, FCM, IFA, FACS	Takizawa et al. (2016)
	Flounder	pAb mAb	WB, FCM, IFA, IHC, FACS WB, FCM, IFA, FACS	Xing et al. (2017a, b) Xing et al. (2020)
	Flounder	mAb	WB, FCM, IFA, FACS	Jung et al. (2020b)
CD8α	Torafugu	pAb	WB, Co-IP, FCM, FACS	Araki et al. (2008)
	Ginbuna crucian carp	mAb	WB, FCM, IFA, FACS	Toda et al. (2009)
	Salmon	mAb	IHC	Hetland et al. (2010)
	Orange-spotted grouper	pAb	WB, FCM, IFA	Chang et al. (2011)
	Trout	mAb	WB, Co-IP, FCM, IFA, IHC, FACS	Takizawa et al. (2011)
	Flounder	mAb	WB, FCM, IFA, FACS	Jung et al. (2021)
CD8β	Flounder	pAb	WB, FCM, IFA, IHC, FACS	Xing et al. (2017a, b)
	Flounder	mAb	WB, FCM, IFA, FACS	Jung et al. (2021)

*Co-IP* Co-immunoprecipitation, *WB* Western blotting, *FCM* Flow cytometry, *IFA* Immunofluorescence assay, *IHC* Immunohistochemistry, *FACS* Fluorescence activated cell sorting

Boardman et al. (2012) prepared a monoclonal antibody against the peptide in the cytoplasmic tail region of CD3ɛ protein in trout (*Oncorhynchus mykiss*) and found that CD3ɛ^+^ cells were most abundant in the thymus and skin but did not co-locate with IgM^+^ cells (Boardman et al. 2012). The antibodies against the intracellular sequences of CD3ɛ chain have been used to identify salmon and trout CD3^+^ T cells in fixed or permeabilized cells (Boardman et al. 2012; Koppang et al. 2010). Furthermore, the extracellular peptides of CD3 molecules were also selected for antibody production in Atlantic salmon and flounder (*Paralichthys olivaceus*), respectively (Jung et al. 2017; Maisey et al. 2016; Tang et al. 2017). In a previously study, flounder CD3^+^ T cells were observed in tissues and no cross-reaction with mIgM^+^ B cells were found. Furthermore, the percentages of CD3^+^ T lymphocytes sharply increased after immunization with inactivated *Edwardsiella tarda* (Tang et al. 2017). Jung et al. (2017) also produced a monoclonal antibody against the CD3ɛ chain in flounder, and the isolated CD3^+^ T cells detected the expression of related genes on T cells, such as TCR, CD4 and CD8 (Jung et al. 2017). CD3^+^ T cells have also been targeted directly with in situ hybridization in fish, and the results showed that fugu CD3 positive cells were detected in kidney, spleen and thymus (Araki et al. 2005).

## αβ and γδ T cells are included in CD3^+^ T lymphocytes

According to the composition of TCR chains, CD3^+^ T lymphocytes can be subdivided into αβ and γδ T cells (Wan et al. 2017). Conventional αβ T cells are the more plentiful T cell type (90–95%), circulating in lymphoid organs and blood, and can be classified as CD4^+^ and CD8^+^ T cells depending on the surface glycoproteins (Hayday 2000; Prinz et al. 2013; Silva-Santos et al. 2015). CD4^+^ and CD8^+^ T cells in teleost fish will be the main focus of attention in the following sections. Among this small group of cells, which represent only 5–10% of T cells, mammalian γδ T cells are the most primitive immune cells and are mainly found in epithelial and mucosal tissues. γδ T cells have been identified and functionally characterized only in zebrafish (Wan et al. 2017). Therefore, further studies on the immune properties of fish γδ T cells and their functional differences with αβ T cells are needed.

## Diversity of CD4 molecules and CD4^+^ T lymphocytes in fish

### Molecular characterization of CD4 molecules

**Fig. 3:**
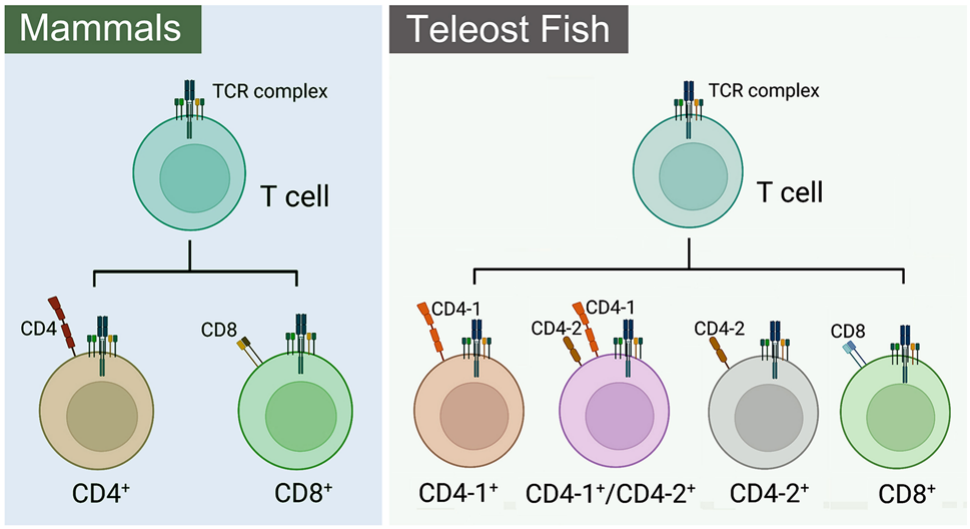
Classification of T cell subsets in mammals and teleost fish. In mammals, there is only one CD4 gene, so based on the expression of CD4 and CD8 co-receptors, T cells can be classified into CD4^+^ and CD8^+^ T cell subsets, where CD4^+^ T cells are functionally known as T helper cells (Th) and CD8^+^ T cells are called cytotoxic T cells (Tc or CTL). CD4^+^ and CD8^+^ T cells were also identified in teleost fish. In contrast, two CD4 genes (CD4-1 and CD4-2) were cloned in fish, and CD4^+^ T cells can be classified into CD4-1 single positive (CD4-1 SP), CD4-1 and CD4-2 double positive (CD4DP) and CD4-2 single positive (CD4-2 SP) cells

CD4, a co-receptor of T cells belonging to the immunoglobulin superfamily (IgSF), has been cloned in many fish species (Ashfaq et al. 2020; Buonocore et al. 2008; Edholm et al. 2007; Kato et al. 2013; Laing et al. 2006; Maisey et al. 2011; Mao et al. 2017; Patel et al. 2009; Sun et al. 2007; Tran et al. 2020). In mammals, a single CD4 gene with four extracellular Ig-like domains (D1–D4) was characterized, whereas two CD4 molecules (CD4-1 and CD4-2) were found in teleost fish (Fig. 3). CD4-1 contains four extracellular Ig-like domains (D1–D4), similar to single CD4 gene in mammals, and a distinct CD4-2 gene that contains two or three Ig-like domains (D1–D2 or D1–D3) (Kato et al. 2013; Takizawa et al. 2016). CD4-1 is present in all teleost fish species studied to date. CD4-like molecules with two Ig-like domains have been identified in rainbow trout, Atlantic salmon, fugu, Atlantic halibut and flounder, whereas CD4-like molecule with three Ig-like domains has only been reported in catfish (Ashfaq et al. 2020; Castro et al. 2011). CD4 molecules show low amino acid identity between fish and higher vertebrates; however, the characteristics of gene structure, splicing patterns, binding motifs and key residues are conserved, and the gene features of CD4 in teleosts have been well documented (reviewed by Ashfaq et al. 2019; Castro et al. 2011). Based on the organization of Ig-like domains in CD4-1 (V-C-V-C) and CD4-2 (V-C or V-C-V), it has been hypothesized that the four-domain CD4 molecule could have arisen by the duplication of an ancestral two-domain (V-C) receptor (Baixeras et al. 1992; Laing and Hansen 2011; Laing et al. 2006; Triebel et al. 1990; Williams et al. 1989). Furthermore, a lamprey CD4-like gene with only two Ig-like domains (V-C) has been reported and thought to be the primordial two Ig-like domain CD4 molecule in vertebrates (Pancer et al. 2004).

Except a long extracellular region, CD4 in mammals contains a transmembrane domain and a critical cytoplasmic tail with a conserved motif (CXC) that interacts with the tyrosine kinase Lck (Kim et al. 2003; Moore et al. 2009; Turner et al. 1990). During antigen recognition, the extracellular D1 and D2 region of CD4 binds to the MHC class II complex expressed on antigen-presenting cells (APCs), and then the cytoplasmic tail of CD4 noncovalently connects with the Lck protein tyrosine kinase, initiating T cell activation (Maisey et al. 2016; Merwe and Davis 2002; Salmond et al. 2010). The cytoplasmic motif was also found in both fish CD4-1 and CD4-2 molecules, and is thought to participate in the activation of T lymphocytes after association with Lck (Moore et al. 2009). This phenomenon suggests that both teleost CD4 coreceptors have a similar function to mammalian CD4 molecules and engage in the development and activation of T cells. Interestingly, the genes encoding CD4 were not found in the Atlantic cod (*Gadus morhua*) and other gadiform species, showing a unique immune system (Malmstrøm et al. 2016; Star et al. 2011). In cobia (*Rachycentron canadum*), the transmembrane domain and CXC motif are not present in the deduced CD4-2b protein, which suggests a difference in CD4-2b activity (Tran et al. 2020). It is noteworthy that this was the first record of soluble CD4 molecules in fish. In conclusion, although CD4 genes have been cloned in multiple fish species, further studies are needed to determine the details of their expression and function in signaling.

### CD4^+^ T lymphocyte subsets in fish

In addition to being a co-receptor in T cells involved in signal transduction, CD4 is also considered as an important surface antigen for identifying Th cells (Wan and Flavell 2009). In fish, recombinant proteins or synthetic peptides were selected and generated as immunogens for the development of monoclonal antibodies or polyclonal antibodies recognizing CD4 molecules (Xing et al. 2017a; Yoon et al. 2015). The function of CD4 in signal transduction has not been elucidated, but there are many studies on the use of CD4 as T cell surface markers to identify CD4^+^ Th cells (Table 1). CD4^+^ T cells were identified in spotted green pufferfish (*Tetraodon nigroviridis*) at the cellular level (Wen et al. 2011). Furthermore, a CD4-2^+^CD25-like^+^Foxp3-like^+^ Treg-like cell population was first identified and functionally characterized in a pufferfish (Wen et al. 2011). Consequently, after the depletion of Treg-like cells, the nonspecific cytotoxic cell (NCC) activity, mixed lymphocyte reaction (MLR) and inflammation in the intestine are all enhanced in pufferfish (Wen et al. 2011). The mAbs (6D1, 9F1 and 2C1) against CD4-1 have been produced in carp (*Carassius auratus langsdorfii*) to trace CD4^+^ T cells (Toda et al. 2011). In addition, many functional studies have been carried out using the mAbs in carp (reviewed by Nakanishi et al. 2015), and it was suggested that CD4^+^ T lymphocytes in carp function as Th cells in mammals (Nayak and Nakanishi 2013; Shibasaki et al. 2010; Somamoto et al. 2014a, b; Yamasaki et al. 2014). In Japanese pufferfish (*Takifugu rubripes*), high-purity CD4^+^ cells were isolated using a specific anti-CD4 antibody, and T cell surface markers, not B cell or macrophage marker genes, were expressed on sorted CD4^+^ T cells (Kono and Korenaga 2013). In addition, the expression of Th1, Th17, and Treg cytokines in Japanese pufferfish CD4^+^ T cells is up-regulated by stimulation after Lipopolysaccharides (LPS) and Polyinosinic-polycytidylic acid (Poly(I:C)), while Th2 cytokines are down-regulated, indicating that these CD4^+^ cells have a similar profile of Th-type cytokine secretion like in mammals (Kono and Korenaga 2013). Similarly, CD4-1^+^ T lymphocytes were identified by using a polyclonal antibody against CD4 molecules in zebrafish (*Danio rerio*) and the results showed that the expression of master transcription factors and cytokines related to Th1 or Th2-type responses were increased after antigen specific stimulation (Yoon et al. 2015). Takizawa et al. (2016) generated mAbs against trout (*Oncorhynchus mykiss*) CD4-1 and CD4-2 molecules and characterized three CD4^+^ leukocytes (CD4-1/CD4-2 double-positive, CD4-2 single-positive T cells and CD4-1 single-positive monocyte/macrophage populations). In the same study, after infection with *Yersinia ruckeri*, CD4^+^ T lymphocytes generated equivalent levels of cytokines relevant to Th1, Th17, and regulatory T cells, and CD4^+^ monocyte/macrophage populations had high phagocytic capacity. CD4-1^+^ subpopulations of T cells in trout were also reported in another study by using polyclonal antibodies against a peptide from the trout CD4-1 sequence (Maisey et al. 2016). Additionally, a long-term CD4-1^+^ T lymphocyte line was established and assessed in trout using IL-15 as a growth factor (Maisey et al. 2016). In a previous study, we identified CD4^+^ T lymphocytes in peripheral blood, spleen and head kidney from flounder, and CD4-1^+^/CD4-2^+^ T lymphocytes were identified in the majority populations among three of CD4^+^ T lymphocyte subsets (Xing et al. 2017a). Flounder CD4^+^ T cells were also found to respond to specific immunostimulants, up-regulated cytokines, and transcription factors of Th subsets (Xing et al. 2020). Similarly, Jung et al. (2020a, b) investigated the cellular immune response of CD4-1^+^ and CD4-2^+^ T cells after infection with viral hemorrhagic septicemia virus (VHSV) and nervous necrosis virus (NNV) in flounder. A polyclonal antibody anti-CD4-1 was also developed in ginbuna crucian carp (*Carassius auratus langsdorfii*) to analyse the immunity of CD4-1^+^ cell in the granulomatous inflammation against mycobacterial infections (Kato et al. 2019).

### Function of CD4^+^ T lymphocyte subsets

**Fig. 4:**
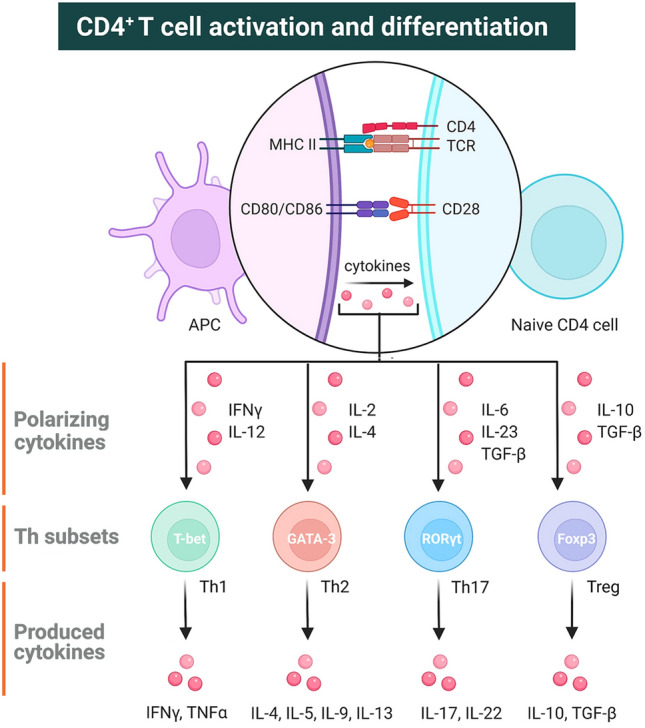
Activation and differentiation of CD4^+^ T lymphocytes. After stimulation by different antigens, signal 1 (binding of the T-cell receptor (TCR) to the peptide-MHCII complex on the antigen-presenting cell (APC) surface) and signal 2 (binding of the T-cell co-receptor CD28 to CD80/CD86 on the APC surface) are required for T-cell activation. The binding of polarizing cytokines to their respective receptor on the T cell surface represents signal 3. Different combinations of these cytokines influence T cell differentiation into distinct effector T cell subtypes (Th1, Th2, Th17, and Treg cells) that produce signature cytokines

Although CD4^+^ T lymphocytes have been extensively characterized in several teleost fish, their precise functions have been only superficially studied. In mammals, CD4^+^ T lymphocytes can differentiate into one of several lineages of Th cell subsets that produce multiple cytokines, which participate in the regulation of inflammation and responses against different pathogens (Zhou et al. 2009; Zhu and Paul 2010; Zhu et al. 2010). In CD4^+^ T cell subsets, there is increasing evidence that the function of Th cell subsets in teleosts is the same as that in mammals (reviewed by Ashfaq et al. 2019; Castro et al. 2011; Fischer et al. 2013; Nakanishi et al. 2015; Tafalla et al. 2016). In a previous study, we found that after the suppression of T lymphocytes, especially CD4^+^ T lymphocytes, the immune responses of B lymphocytes were distinctly inhibited, which suggests that CD4^+^ T lymphocytes regulate the immune response of mIgM^+^ B cells in flounder (Xing et al. 2017b, 2019b). In other fish species, CD4^+^ T cells were also involved in a variety of immune functions, such as stimulating macrophages to increase microbicidal activity, B lymphocytes to produce antibodies and enhancing cell-mediated immunity (Nakanishi et al. 2015). In addition, the genes that encode unique transcription factors and hallmark cytokines of helper T cell subsets are represented in most teleost genomes (Wang et al. 2010). There are ample evidences that CD4^+^ T lymphocytes up-regulate the expression of master transcription factors and cytokines relevant to Th-type responses following antigen specific stimulation in fish (Kono and Korenaga 2013; Takizawa et al. 2016; Tian et al. 2021; Xing et al. 2020; Yoon et al. 2015). These studies support the potential existence of effector T cell subsets in fish (Fig. 4). However, functional studies on CD4^+^ T lymphocytes are far from sufficient and it is difficult to detect CD4^+^ T lymphocytes biased toward a specific Th phenotype in fish. In mammals, CD4 is also found on the surface of other leukocytes, for example, human and rat monocytes/macrophages, but not on monocytes/macrophages from mice and birds (Chan et al. 1988; Gordon and Taylor 2005). Similarly, CD4-1^+^ myeloid cell populations with the highest recorded phagocytic activity and capacity were described in trout (Takizawa et al. 2016), CD4-1 and CD4-2 genes in zebrafish were found not only in lymphocytes but also in monocytes/macrophages and even precursor cells (Yoon et al. 2015). In a previous study, we also found that sorted flounder CD4^+^ cells express CSF-1R, a surface marker of monocytes/macrophages (Xing et al. 2020). In salmon, it has not been well explained that cd8α, cd8β and IgM genes were expressed in sorted CD4^+^ cells (Maisey et al. 2016). Furthermore, it has been shown that the CD4^+^ T cells in carp (*Carassius auratus langsdorfii*) have strong direct antibacterial activity (Nayak and Nakanishi 2013). In mammals, CD4^+^ Tc cells have also been investigated, and they were found to function using the same cytolytic pathways as those in CD8^+^ Tc cells (Appay et al. 2002; Hashimoto et al. 2019; Stalder et al. 1994; Williams and Engelhard 1996). However, the precise roles of CD4^+^ Tc cells remain unclear. Interestingly, studies on the expression characteristics of catfish CD4 molecules showed that two CD4 genes were found on the cytotoxic cell line TS32.17, indicating that in addition to the conventional CD8^+^ Tc cells and CD4^+^ Th cells, CD4^+^ Tc cells may be present in teleost fish (Edholm et al. 2007). These findings show that the characteristics and functions of CD4^+^ cells in different fish species need to be further investigated.

## CD8 function as surface marker of cytotoxic T cells in fish

### Molecular characterization of CD8 molecules

CD8 is a membrane-bound extracellular receptor consisting of an αα homodimer or an αβ heterodimer (Janeway 1992; Zamoyska 1994). CD8α and CD8β consist of an IgV-like extracellular domain, a transmembrane domain and a short cytoplasmic tail, with the α and β peptides linked by disulfide bonds (Veilette et al. 1988). The heterodimer of CD8 is mostly found on mature cytotoxic T cells and thymocytes, while the homodimer is expressed on NK cells, dendritic cells (DCs) and γδ T cells (Lin et al. 1994; Terabe et al. 2008). Two of the CD8 chains have been cloned in multiple teleost species, such as rainbow trout, ginbuna crucian carp, fugu, sea bass, Atlantic salmon and flounder (Hansen and Strassburger 2000; Kato et al. 2013; Maisey et al. 2011; Moore et al. 2005; Picchietti et al. 2009; Somamoto et al. 2005; Suetake et al. 2007). CD8^+^ cells have been functionally identified as Tc cells in teleosts and express a heterodimer of CD8 consisting of α and β chains, as occurs in mammals (Fischer et al. 2006; Nakanishi et al. 2002, 2011; Somamoto et al. 2014a, b). In mammals, a conserved binding motif p56^Lck^ is present in the cytoplasmic tail of CD8α, whereas it is not found in the CD8β chain. In contrast, both CD8α and CD8β genes in fish contain the binding motifs, suggesting that CD8 molecules may have signaling functions in the heterodimers and homodimers of teleosts (Quiniou et al. 2011; Tafalla et al. 2016). To date, nothing is known about the differences between homodimers and heterodimers of fish CD8 molecules and their functions as co-receptors of T cells.

### Identification of CD8^+^ T lymphocytes

T cytotoxic (Tc) cells express CD8 chains involved in the interaction with peptide-MHC class I, and Tc cells were thus identified by detecting CD8 anitgens using specific antibodies (Araki et al. 2008). Currently, several antibodies recognizing fish CD8 molecules have been generated (Table 1), and the functions of T cytotoxic cells in fish are outlined in some reviews (Fischer et al. 2006; Nakanishi et al. 2002, 2011; Somamoto et al. 2014a, b). CD8α^+^ leukocytes from fugu (*Takifugu rubripes*) were characterized by using anti-CD8α antiserum generated in mouse by DNA-immunization (Araki et al. 2008). The CD8α and CD8β genes were expressed in sorted fugu CD8α^+^ leukocytes, whereas CD4 and immunoglobulin light chain (IgL) genes were detected only in CD8α^−^ cells. In addition, fugu CD8α^+^ leukocytes gave a response to PHA but not to LPS, suggesting that teleost CD8^+^ cells have characteristics similar to mammalian CD8^+^ T lymphocytes. In ginbuna crucian carp, it was demonstrated for the first time that CD8^+^ lymphocytes were the principal cells participating in specific cell-mediated cytotoxicity against allogeneic targets in fish, as reported in higher vertebrates (Toda et al. 2009). Shibasaki et al. (2010) claimed that donor-derived CD8α^+^ T lymphocytes in carp play critical roles in the response to acute graft-versus-host reaction as in mammals. Furthermore, the protective immunity against intracellular pathogen infection and direct antibacterial activity of CD8α^+^ T cells have been demonstrated in carp (Nayak and Nakanishi 2013; Yamasaki et al. 2014). After challenge with infectious salmon anaemia virus (ISAV), salmon CD8-labeled cells participated in the early activation of cellular immunity in the defense against ISAV (Hetland et al. 2010). In orange spotted grouper (*Epinephelus coioides*), the effector or target cells were obtained from, and the MHC class I restriction and specific cytotoxicity of CD8^+^ cells were measured in, the same individual fish (Chang et al. 2011). The results showed that grouper cytotoxic CD8^+^ cells have heterogeneous features in terms of specific antigen recognition and MHC I restriction (Chang et al. 2011). CD8α^+^ T cells have also been detected in trout by using mAbs and it was found that high abundances of CD8^+^ cells are present in the thymus, intestine and gills, but low abundances in the spleen, pronephros and blood (Takizawa et al. 2011). After stimulation with PHA, trout CD8^+^ cells up-regulated the Tc cells effector genes, such as perforin, granzyme and IFN-γ, which suggests the functions of teleost CD8^+^ T lymphocytes are similar to those in mammals (Takizawa et al. 2011). In a previous study, where flounder CD8^+^ lymphocytes were identified in the peripheral blood, spleen and head kidney by using an antiserum reaction to the CD8β chain, it was found that there is no cross-reaction between CD8β^+^ and CD4^+^ lymphocytes (Xing et al. 2017a). After hirame novirhabdovirus (HIRRV) infection or immunization, the ratios of CD8^+^ T cells increased more rapidly than CD4^+^ T cells, which indicates that CD8^+^ T cells play main roles in the response to HIRRV (Xing et al. 2018a). Similarly, Jung et al. (2021) also found that flounder CD8^+^ T cells were mainly involved in the adaptive immune response against viruses. The antiviral functions of CD8^+^ T cells in teleost fish were reviewed by Somamoto et al. (2014a, b).

### CD8α^+^ DC-like cells in fish

It has been shown that flounder CD8^+^ T lymphocytes proliferate after immunization with a DNA vaccine (Xing et al. 2019a). Furthermore, studies in mammals have shown that CD8^+^ T lymphocytes can enhance the protective effect of vaccines after immunization with DNA plasmids (Ulmer and Otten 2000). These results suggest that the immune response of fish CD8^+^ T cells can also be an important evaluation indicator of the efficacy of DNA vaccines. Interestingly, mammalian CD8α can be expressed as CD8αα homodimers on different leukocytes, such as αβ or γδ T cells, NK cells, DCs, regulatory T cells and even macrophages (Addison et al. 2005; Bonneville and Lang 2002). In fugu, the expression of CD8α has also been detected in monocytes/macrophages (Araki et al. 2008). Recent studies have shown that a DC-like subpopulation co-expressing CD8α and MHC II on the surface of cells in the skin, gills, gut and olfactory organs of rainbow trout (*Oncorhynchus mykiss*) (Granja et al. 2015; Sepahi et al. 2016; Soleto et al. 2018, 2019). These results provide the evidence for the existence of CD8α^+^ DCs in non-immune tissue of teleost fish, and support the hypothesis that all mammalian cross-presenting DCs have a common origin. Due to the lack of sufficient mAbs against the CD8β chain in many fish species, it remains unknown whether CD8αα^+^ and CD8αβ^+^ lymphocyte subpopulations have different functions. Hence, further studies are needed to produce specific antibodies against both CD8α and CD8β chains in different teleost fish.

## Other CD antigens expressed on T cells

### CD28 and CTLA-4

There are several other pivotal surface-associated molecules involved in the activation, proliferation and differentiation of T cells, such as CD28, cytotoxic T lymphocyte antigen 4 (CTLA-4/CD152), CD40L (CD154) and CD2. According to the mammalian paradigm, activation of T cells requires the following two sets of signals: The first signal is the delivery of processed antigen to the TCR via MHC molecules; the second is known as the costimulatory signal, which is delivered to receptors on T cells via costimulatory molecules (Hu et al. 2012; Paterson et al. 2009; Rudd et al. 2009). The co-stimulatory receptors CD28 and CTLA-4 of T cells are known to play essential roles in transmitting the second signal. CD28 and CTLA-4 have reverse effects on T cell stimulation, the activation of TCR is enhanced by CD28 but inhibited by CTLA-4 (Chen and Flies 2013). In mammals, CD28 and CTLA-4 are transmembrane protein members belonging to the IgSF, and they both interact with the same ligands, i.e., members of the B7 family, CD80 (B7-1) and CD86 (B7-2) expressed on APCs (Esensten et al. 2016; Sansom 2010). Studies have shown that both CD28 and CTLA-4 homologs are conserved in fish and the pathway of CD28-CD80/86 may present in teleosts (Bernard et al. 2006, 2007; Fang et al. 2018; González-Fernández et al. 2021; Hu et al. 2012; Huang et al. 2018; Jeswin et al. 2017; Sugamata et al. 2009; Zhang et al. 2009, 2018). The features of CD28/CTLA-4 in teleosts were discussed in depth in two reviews (Castro et al. 2011; Laing and Hansen 2011).

### CD40L

CD40L (also known as CD154) is a 39-kDa glycoprotein of the TNF family that is initially found on activated CD4^+^ T cells (Graf et al. 1992; Kooten and Banchereau 2000). The natural receptor for CD40L is the type I membrane-bound protein CD40, which was originally identified as a surface antigen expressed on mature and activated B lymphocytes (Clark 1990). In mammals, it has been shown that the CD40-CD40L mediated contact-dependent signal is essential for Th-dependent B lymphocyte proliferation, Ig production and type switching, and even memory responses (Castigli et al. 1994; Foy et al. 1993; Kawabe et al. 1994; Renshaw et al. 1994; Xu et al. 1994). CD40-CD40L interactions also play an important role in the functional communication between T lymphocytes and DCs (Caux et al. 1994; Cella et al. 1996). Furthermore, in mammals, CD40L-CD40 interactions direct T lymphocyte maturation towards the Th1 phenotype through the induction of proinflammatory cytokines (Mackey et al. 1998; Pinchuk et al. 1994). In fish, CD40 and CD40L genes were cloned in various of fish species, such as flounder (*Paralichthys olivaceus*), trout (*Oncorhynchus mykiss*), zebrafish (*Danio rerio*), Atlantic salmon (*Salmo salar*) and humphead snapper (*Lutjanus sanguineus*) (Cai et al. 2017; Glenney and Wiens 2007; Gong et al. 2009; Lagos et al. 2012; Park et al. 2005). In zebrafish, the expression of CD40L was inhibited by cyclosporin A, and the production of IgM was affected by the supplement of anti-CD40L or soluble CD40 (Gong et al. 2009). These results provide evidence for the existence of a CD40-CD40L mediated costimulatory pathway in fish. Interestingly, Xing et al. (2018b) showed that CD40 was not only expressed on sIgM^+^ B lymphocytes, but also on CD4^+^ and CD8^+^ T subsets. In mammals, increasing evidence confirms the presence of CD40 molecules on T cells, and CD40 may act on both CD4 and CD8 T lymphocytes (Munroe and Bishop 2007; Munroe 2009). However, further studies are needed to elucidate the exact role of CD40 in fish T cells.

### CD2

CD2 (lymphocyte function-associated antigen-2) is a cell adhesion molecule expressed on all mature peripheral blood T cells, thymocytes and natural killer (NK) cells (Davis and van der Merwe 1996; Seed and Aruffo 1987; Springer et al. 1987). Its ligand, CD58, which is also an adhesion molecule, is expressed on hemopoietic and non-hemopoietic lineages such as DCs, macrophages, erythrocytes, and endothelial cells (Dengler et al. 1992; Karmann et al. 1996; Moingeon et al. 1989; Ocklind et al. 1992). Functionally, the interaction of CD58 with CD2 plays an important role in the adhesion between the T cells and APCs and also provides the optimal spacing for the antigen recognition of TCR (Cheadle et al. 2012; Zhu et al. 2006). CD2 was identified in channel catfish and zebrafish, and the interaction of CD58 with CD2 has been well demonstrated in zebrafish where it provides a primary costimulatory signal for the complete activation of CD4^+^ T cells in adaptive humoral immunity (Shao et al. 2018; Taylor et al. 2015).

## Summary and perspectives

In conclusion, a variety of CD molecules exist on T cells in fish, and in this review, we described the role of CD molecules as surface markers in the identification of teleost T lymphocyte subpopulations. It is undeniable that some CD antigens, which are the surface markers of T cells, are also expressed on other cell lineages in fish. At present, identification and characterization of CD molecules on fish T cells are far from sufficient, and antibodies for the clear discrimination of Th and Tc subsets according to the CD antigens of mammals are urgently needed for multiple fish species. There is a lack of direct evidence about the precise role of CD molecules in T cell activation and signaling from fish, and more studies are needed to support these hypotheses. In addition, the effectors of Tc (perforin and granzyme) should be well characterized, which can give insights into the killing mechanism of CD8^+^ Tc in fish. The definition of different phenotypical CD4^+^ Th subsets in fish still needs more evidences, and multiple cytokines and transcription factors involved in Th-type immunity should be functionally characterized. Furthermore, the mechanisms by which fish CD4^+^ Th cells activate B cells, CD8^+^ T cells and macrophages need to be elucidated. The antibodies against critical cytokines, such as IFN-γ, IL-2, IL-4 and IL-17, should be produced as they will be powerful tools to detect the preference of cytokine secretion by CD4^+^ T cells, and meaningful for the differentiation of CD4^+^ T cells in fish. Thus, there is still an urgent need to accurately delineate the pathways of T cell response to vaccination as well as how the fish T cell subsets are able to regulate a protective response. In combination, such knowledge would deepen our understanding about the role of fish T lymphocyte subsets in adaptive immunity and facilitate the health management and development of vaccines in aquaculture.

## Data Availability

All data generated or analyzed during this study are included in the manuscript.
